# Construction Notes

**Published:** 1907-03-23

**Authors:** 


### CONSTRUCTION NOTES.

### CARDIFF INFIRMARY.

The supplementary report of the Cardiff Infirmary makes a forcible appeal for £30,000 for erecting a new pavilion, new gynæcological operating-room, corridor, and lifts, and a clinical laboratory, museum, and new post-mortem room. The approximate cost of these buildings, including lighting, heating, and furniture, is estimated to be as follows: Pavilion for 64 beds, plus six beds for isolation purposes, including the nurses' and servants' bedrooms, £22,500; new gynæcological operating-room, £2,500; corridor and lifts to connect the wards on the first floor, £2,000; clinical laboratory, museum, and new post-mortem room, £3,000; total, £30,000.

### TAUNTON AND SOMERSET HOSPITAL.

The success of the great structural improvement scheme inaugurated six years ago by the Committee of Management of the Taunton and Somerset Hospital is something upon which the hospital Executive and all concerned may be deservedly congratulated. £11,663 has been expended in carrying out the various improvements, which include, in addition to those comprised in the original estimates, a new disinfector and the enlargement of the disinfecting chamber, at a cost of £200. At the annual meeting the President, Mr. Portman, was in the happy position of being able to announce that the deficit on the building fund is now only £94, so handsomely have the appeals of the committee been responded to.

### WIGAN ROYAL ALBERT EDWARD INFIRMARY.

This institution is appealing for funds to complete the necessary extension scheme. A sum of £25,000 is needed to provide further accommodation. Since 1893 nearly £16,000 has been spent on necessary improvements. These include a new mortuary, mortuary chapel, and pathological department; a new dining hall for the nurses and probationers; sanitary improvements and drainage; provision of an adequate hot water supply for bathrooms, kitchens, and for domestic purposes, also an improved heating apparatus; alteration and enlargement of the operating theatre; building a boundary wall and costs of transfer of land; structural alterations and improvements in the kitchens and laundry, including the building of workshops; new ward floors; fire-escapes outside landings and iron staircases; and finally a separate hospital for isolation cases. Donations amounting to nearly £2,000 have already been promised.

### VICTORIA HOSPITAL, BLACKPOOL.

The thirteenth annual report of the Victoria Hospital records the satisfactory progress made with the scheme for the extension of the institution. The Board, during the year, decided to carry out the complete extensions, rather than enlarge the administrative portion only, particularly as the improvement of the ward for females is much needed. The aggregate cost of these extensions, for which contracts have been entered into, is estimated at £14,590. These extensions comprise the enlargement of the two wards, provision of accommodation for the out-patients' department, the enlargement of the administrative block, a new operating theatre, a more capacious laundry, and many other additions and alterations, all of which are found absolutely necessary for the efficient and economical working of the institution. After providing for the contract sums for all the extensions for which in the year 1903 an appeal was made to the public, there is an estimated deficiency of about £3,869. These extensions will make it desirable to abandon the temporary iron wards erected in 1898. They have served the purpose for which they were erected, and the Board purpose offering them for sale as soon as possible.

### LITTLEBOROUGH JOINT ISOLATION HOSPITAL.

The new isolation hospital for Littleborough, Wardle, and Milnrow is situated close to Hollingworth Lake, on an elevated site 580 feet above sea-level.

The hospital buildings have been arranged on the pavilion system, and consist of the administration block, scarlet fever pavilion, observation pavilion, laundry, disinfecting block, and porter's lodge. The administration block is situate about 60 feet from the boundary wall, and is approached from the entrance gates by a wide avenue. The elevations of this block are of a pleasing design, faced with Newhey red bricks and Yorkshire stone dressings. The accommodation provided on the ground floor is: Entrance vestibule and large hall, with staircase to the upper floor, matron's sitting-room with stores adjoining, nurses' sitting and dining-room, doctor's room and dispensary with lavatory, etc., attached. From the hall extends a corridor, on one side of which is placed a tradesmen's entrance, and at the end is the kitchen, with scullery, pantries, and stores, from which access is obtained to an enclosed yard. An entrance has also been provided from the grounds at a point near the ward blocks for the use of the nurses going to and from their duties, and adjacent to this a room has been arranged in which the nurses will change their clothing when coming from duty before entering the administration block. On the upper floor bedrooms have been provided for the matron, seven nurses, and for female servants, with bathroom and other necessary accommodation, and several large linen stores.

The scarlet fever pavilion is situated on the north side, some 140 feet away, and the observation pavilion is situated on the west, 70 feet distant. Each of the ward blocks has its main entrances in the centre of the front elevations, and is thus divided into two wards for the different sexes, each ward in the scarlet fever block containing six beds, and the observation wards two beds each, with space for a third bed if required, thus providing present accommodation for eighteen beds. When the enteric fever pavilion, of similar capacity to the scarlet fever block, is erected, hospital accommodation will be provided for thirty beds. Adjacent to the entrance in the centre between the wards in each block a room for the nurses on duty has been provided, and at each end are retiring and bath-rooms, etc. Ample clothing and other necessary stores have also been arranged.

The heating of the wards is by means of Shorland's heating and ventilating stoves, which have been provided and fixed by the firm referred to, and the sanitary fittings throughout the hospital have been supplied by Messrs. Morrison, Ingram and Company, of Manchester. The floors of the wards are of concrete and maple, with ample air-space beneath for ventilation between the sub-soil and the floor. The walls are plastered with special mortar, finished smooth and painted and varnished, and the woodwork has also been painted and varnished throughout, great care having been taken to make the premises as sanitary as possible. On the front of each of the wards there has been arranged a terrace with a verandah covering, thus affording opportunity for such patients as are able to take it, of outdoor exercise under cover.

The hospital has been designed by, and erected under, the supervision of Messrs. J. H. Andrews and Butterworth, architects, of 78 King Street, Manchester, and the contractors for the whole of the work are Messrs. Dryland and Preston, of Littleborough.

